# Evolutionary analysis of DELLA proteins in sweet potato and related species reveals their roles in development and stress responses

**DOI:** 10.3389/fpls.2025.1494621

**Published:** 2025-01-23

**Authors:** Zhidan Zuo, Haoqiang Zhao, Yue Fan, Yixuan Zhu, Wenpeng Song, Hong Zhai, Shaozhen He, Huan Zhang, Ning Zhao, Qingchang Liu, Shaopei Gao

**Affiliations:** Key Laboratory of Sweet Potato Biology and Biotechnology of Ministry of Agriculture and Rural Affairs, College of Agronomy and Biotechnology, China Agricultural University, Beijing, China

**Keywords:** *Ipomoea* species, sweet potato, DELLA gene family, abiotic stress, evolutionary analysis

## Abstract

DELLA proteins act as master negative regulators in the gibberellin signaling pathway, which controls numerous aspects of plant growth and development. Despite the pivotal role of DELLA proteins, a comprehensive genome-wide analysis of the *DELLA* gene family in sweet potato (*Ipomoea batatas*) and its related species has yet to be conducted. Here, we performed a comparative analysis of this gene family among six *Ipomoea* species, including *Ipomoea batatas*, *Ipomoea trifida*, *Ipomoea triloba*, *Ipomoea nil*, *Ipomoea cairica*, and *Ipomoea aquatica*. Among the six *Ipomoea* species, only *I. nil* contains five *DELLA* genes, while the remaining species have three *DELLA* genes each. The *DELLA* genes were categorized into three distinct subgroups based on the phylogenetic topology in selected *Ipomoea* species. Comparative analysis of gene structure and protein motifs revealed that members within the same phylogenetic group exhibit comparable exon/intron and motif organization. The *cis*-regulatory elements of the *DELLA* gene in selected *Ipomoea* species contain unique promoter elements, indicating the presence of species-specific regulatory mechanisms. A multitude of shared *cis*-regulatory elements related to stress responses were identified in the *DELLA* gene promoters. Furthermore, a syntenic analysis indicates two groups of syntenic *DELLA* genes have undergone several rearrangements. The results of the duplication analysis indicated that dispersed duplications contribute to the expansion of the *DELLA* genes. Moreover, the *DELLA* genes in sweet potato display an expression pattern that tends to control the growth and development of either the aerial or below-ground parts, and they are responsive to a range of hormones and abiotic stresses. Thus, these findings provide insights into the evolutionary history of *DELLA* genes within the genus *Ipomoea* and the functions of sweet potato *DELLA* genes.

## Introduction

DELLA proteins are a subgroup of the GRAS family of transcription factors that are unique to plants. They are important regulators in various aspects of plant growth and development, including seed germination, stem elongation, leaf development, flowering time, and responses to environmental stresses (Davière and Achard, 2013; Olszewski et al., 2002; Peng and Harberd, 2002). They function as key negative regulators in the gibberellin (GA) signaling pathway, inhibiting plant growth by repressing the expression of growth-related genes (Sarwar et al., 2023). The DELLA domain, situated near the N-terminus of the protein, is a signature domain of the DELLA protein family and a crucial region for interaction with the GA receptor GID1. In the absence of GA, DELLA proteins accumulate and bind to growth-promoting factors, thereby inhibiting their transcriptional activity and consequently repressing the expression of growth-related genes. This results in the dwarfing of the plant (Shani et al., 2024). Conversely, in the presence of GA, the receptor GID1 binds to it, thereby facilitating the interaction between GID1 and DELLA proteins. The GID1-GA-DELLA complex is recruited to the SCFSLY1/GID2 E3 ubiquitin ligase complex, which catalyzes the ubiquitination and subsequent degradation of DELLA proteins, thereby relieving their inhibitory effect on growth. It is noteworthy that DELLA proteins are not solely involved in the GA signaling pathway; they also interact with other hormone signaling pathways, including auxin, brassinosteroids, and abscisic acid, as well as environmental signaling pathways, such as light, temperature, and nutrients (Fu and Harberd, 2003; Gao et al., 2011; Hou et al., 2010; Li et al., 2015, 2016). These interactions allow DELLA proteins to integrate diverse signals, thereby enabling precise regulation of plant growth and development (Degefu and Tesema, 2020; Lozano-Juste and León, 2011).

The origin of DELLA proteins can be traced back to the early stages of mosses and ferns, with the conserved domains of these proteins first evolving in ferns (Kato et al., 1962; Radley, 1961). As the terrestrial plants evolved, the DELLA proteins underwent a gradual process of evolution, resulting in the emergence of a mechanism enabling interaction with the gibberellin receptor GID1. This interaction was initially observed in ferns, rather than in mosses, suggesting an independent origin during the evolution of land plants. The GA-DELLA-GID1 signaling pathway was gradually formed in later plants (Gao et al., 2008; Yasumura et al., 2007). In vascular plants, the *DELLA* gene family underwent gene duplication, resulting in the emergence of multiple *DELLA* genes. This has led to considerable diversity in the number and expression patterns of *DELLA* genes in different species (Aya et al., 2011; Hirano et al., 2007). For example, the *DELLA* gene family in Arabidopsis consists of five members: *GA insensitive* (*GAI*), *Repressor of ga1-3* (*RGA*), *RGA-like 1* (*RGL1*), *RGA-like 2* (*RGL2*), and *RGA-like 3* (*RGL3*), while monocot species typically contain a single *DELLA* gene, such as *SLR1* in rice and *RHT-1* in wheat (Ikeda et al., 2001; Peng et al., 1999). The evolution of DELLA proteins is characterized by changes in their structure and function. These reflect the complex mechanisms that plants use to adapt to their environment and to regulate growth and development. DELLA proteins are highly conserved among terrestrial plants, ranging from mosses to angiosperms. This highlights the pivotal role that these proteins have played in the evolutionary history of plants.

*Ipomoea* is the largest genus in the family Convolvulaceae, with 600-700 species (Austin and Huáman, 1996). *Ipomoea* species are distributed globally and of great significant agricultural and industrial importance (Liu, 2017; Morita and Hoshino, 2018; Si et al., 2023; Sun et al., 2022). Water spinach (*I. aquatica*) is a widely cultivated vegetable known for its high nutritional value and potential medicinal applications (Malakar and Choudhury, 2015). *I. cairica* is often utilized as a ground cover plant, aiding in soil stabilization and erosion prevention (Srivastava and Shukla, 2015). The flowers of Japanese morning glory (*I. nil*) are highly prized for their ornamental value and used widely in landscaping and beautification projects (Hoshino et al., 2016). *I. trifida* and *I. triloba* are regarded as close wild relatives of the sweet potato, providing vital reference points for the study of sweet potato evolution and breeding (Hirakawa et al., 2015; Li et al., 2019; Wu et al., 2018). Sweet potato (*I. batatas*), a vital food and industrial crop, possesses storage roots that are rich in carbohydrates, vitamins, and minerals. Furthermore, the stems and leaves of this plant can also be used as animal feed (Bovell-Benjamin, 2007; Guo et al., 2024; Ji et al., 2015). In addition to its nutritional value, sweet potato is a valuable industrial raw material used in the production of starch, alcohol, and biofuels. This makes it an important source of materials for industrial applications (Bach et al., 2021; Odebode et al., 2008).

To date, there are no reports on the comparative genomics of *DELLA* in *I. trifida*, *I. triloba*, *I. nil, I. cairica*, *I. aquatica* and *I. batatas*. The evolutionary dynamics of *DELLA* genes in *Ipomoea* species remains unclear. This study provides a comprehensive comparative analysis of *DELLA* genes in six *Ipomoea* species, focusing on their evolutionary history, structural features, and functional diversification. The aim of this study is to identify genetic targets that could be used for the genetic improvement of sweet potato.

## Materials and methods

### Identification of *DELLA* genes in *Ipomoea* species

The complete genome sequences of *I. aquatica*, *I. cairica*, *I. nil*, *I. triloba*, *I. trifida*, *I. batatas* were obtained from National Genomics Data Center (NGDC) (https://ngdc.cncb.ac.cn/gwh/Assembly/986/show), the Zenodo repository (https://zenodo.org/records/6792002#.Y90Mb3ZBy4Q), the Shigen database (http://viewer.shigen.info/asagao/index.php), and the Ipomoea Genome Hub (https://sweetpotao.com/). To achieve a more comprehensive identification of *DELLA* genes, the BLAST algorithm and the Hidden Markov Model (HMM) were employed to predict the genes. The amino acid sequences of *DELLA* genes from the *I. batatas*, identified by previous study as queries (BLASTP, E value ≤ 1×10−5), were used in the identification of *DELLA* genes. Subsequently, all putative *DELLA* genes were validated using the CD-Search tool (https://www.ncbi.nlm.nih.gov/Structure/cdd/wrpsb.cgi). Then, the remaining *DELLA* proteins were numbered in accordance with their degree of similarity to the sweet potato *DELLA* protein.

### Subcellular localization and signal peptide prediction

Subcellular localization prediction was performed using the DeepLoc 2.1 server (https://services.healthtech.dtu.dk/services/DeepLoc-2.1/). Furthermore, the ExPASy tool (https://web.expasy.org/protparam/) was used to analyze the physicochemical parameters of the identified *DELLA* proteins.

### Phylogenetic and evolutionary analysis

Phylogenetic analysis of *DELLA* genes from different species was performed using MAFFT with the default parameters (Katoh et al., 2005). The results of multiple sequence alignment were then trimmed using the TrimAl software (Capella-Gutiérrez et al., 2009). Subsequently, the phylogenetic tree was constructed using the Iqtree2 software with 1000 bootstrap replicates, which can automatically select the best model (Minh et al., 2020). The phylogenetic trees were visualized using the Interactive Tree of Life (ITOL) platform (https://itol.embl.de/index.shtml).

### Analysis of the conserved protein motifs and gene structure

The conserved motif of DELLA proteins was analyzed using Multiple Em for Motif Elicitation (MEME, https://meme-suite.org/meme/tools/meme), which identified 10 motifs using the classical discovery mode. The gene structure was visualized using the GSDC (http://gsds.gao-lab.org/).

### *Cis*-element identification in the promoter

The 2000 bp sequence upstream of the translation start sites of the *DELLA* genes was extracted using the TBtools software (Chen et al., 2023). The PlantCARE tool (http://bioinformatics.psb.ugent.be/webtools/plantcare/html/) was employed for the analysis of the *DELLA* gene promoters. Visualization of the *cis*-elements was conducted using the TBtools software and the Python package Seaborn.

### Prediction of protein secondary and three-dimensional structures

The secondary structure of the DELLA protein was predicted by NetSurfP-3.0 (Høie et al., 2022) and the three-dimensional (3-D) structures were predicted by the AlphaFold3 (Abramson et al., 2024). The 3-D structure of the DELLA domain was illustrated in PyMOL (DeLano, 2002).

### Syntenic analysis and classification of gene duplication

The BLASTP result was subjected to analysis by MCScanX, which generated the collinearity blocks across the entire genome (Wang et al., 2012). The collinearity pairs were extracted and a collinearity map was generated using the CIRCOS software with modified parameters (https://circos.ca/documentation/course/). The duplicate_gene_classifier script of MCScanX was used to analysis the classification of gene duplications.

### Expression analysis and gene interaction network construction

To analyze the expression of *DELLA* genes in sweet potato, publicly available RNA sequencing (RNA-Seq) based expression data under the BioProject accession numbers PRJCA000640 and PRJNA511028 in National Genomics Data were utilized. The RNA-Seq dataset covers a range of sweet potato tissues. Firstly, Fastp was used to remove the adapt and low-quality reads (Chen et al., 2018). Second, the resulting reads were mapped to the sweet potato reference genome using STAR (Dobin et al., 2013). Finally, Featurecount was used to obtain the exon abundance matrix (Liao et al., 2014). In-house python script was used to calculate the transcripts per kilobase of the exon model per million mapped reads (TPM) values for the *DELLA* genes. The multiple tissue expression profiles of sweet potato were normalized using the log2 (TPM+1) transformation. Finally, a heatmap was generated using the seaborn package in Python, based on the above normalized expression values. The String database was used to predict genome-wide gene interactions in sweet potato.

The expression of *DELLA* genes in sweet potato was confirmed by reverse transcription quantitative PCR (RT-qPCR). In vitro-grown sweet potato seedlings were cultured on Murashige & Skoog (MS) medium at 27 ± 1°C under a photoperiod consisting of 13 h : 11 h, light : dark (cool-white fluorescent light at 54 μmol m−2 s−1), then the 4-week-old sweet potato seedlings were treated with 100 μM ABA or 20% polyethylene glycol (PEG) 6000 for 12 h, respectively. Total RNA was extracted from samples of seedlings treated with the TRIzon reagent (CWBIO, Taizhou, China). First-strand cDNA was synthesized using a PrimeScript RT reagent Kit (TaKaRa, Dalian, China). StepOne PLus Real-Time PCR system (Applied Biosystems) with SYBR green assays was used for RT-qPCR analysis. Primers and reference gene information for RT-qPCR are listed in **Supplementary Table S2**. All experiments were performed in three biological repeats. The relative expression of genes was calculated using the 2^−ΔΔCT^ method. Analysis for statistical significance was performed using least significant difference (LSD) test and two-tailed Student’s *t*-test in Python, *P* value < 0.05 for significance.

### Subcellular localization analysis

Subcellular localization analysis was conducted using the *DELLA* genes in sweet potato. The coding sequence (CDS) without stop codon was cloned into the pCAMBIA2300-35S::eGFP (C) vector, which contained a 35S-driven enhanced green fluorescent protein (eGFP). The In-Fusion cloning kit named KunGre ™ II Multi One Step Cloning Kit, which is produced by Beijing Greact Biotechnology, was used to clone. KpnI and BamHI were used as the restriction sites of pCAMBIA2300-35S::eGFP (C) vector. The fusion constructs and empty vector were introduced into *Agrobacterium tumefaciens* strain EHA105, respectively. Agrobacterium-mediated transient expression system in the leaves of tobacco (*Nicotiana benthamiana*) as described previously (Xue et al., 2022). After 8 h of dark culture, the transformed tobacco was cultured normally (dark for 8 h at 22°C, light for 16 h at 24°C). After 48 h, the LSM900 confocal laser scanning microscope (Zeiss, Jena, Germany) was used to observe the GFP fluorescence signals. The primers used in this study are listed in **Supplementary Table S3**.

## Results

### Identification of the *DELLA* genes in the six *Ipomoea* species

**Figure 1:**
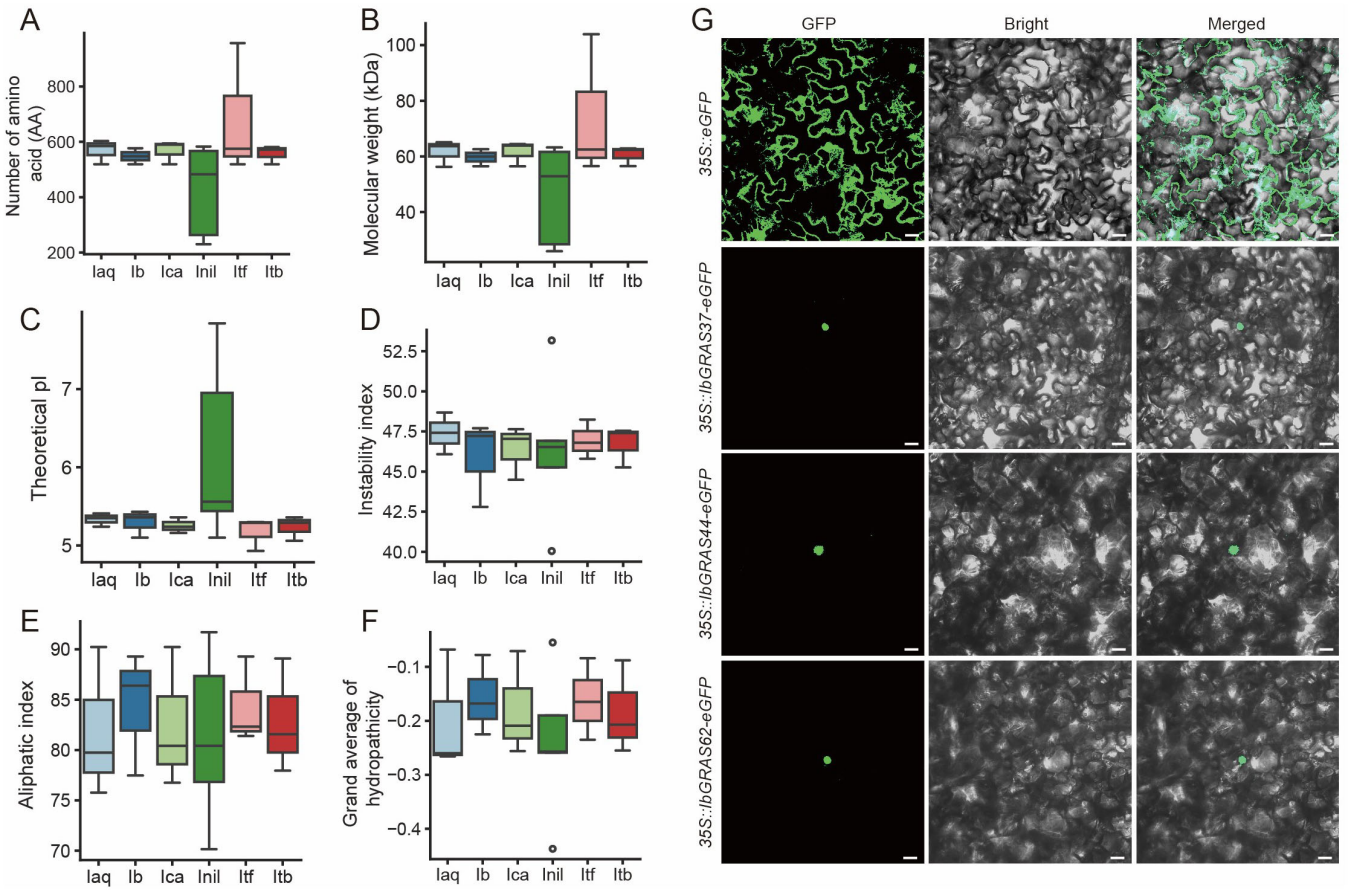
Physicochemical properties of all identified DELLA proteins in six *Ipomoea* species. **(A)** Protein length; **(B)** Molecular weight (MW); **(C)** Isoelectric point (pI); **(D)** Instability index; **(E)** Aliphatic index; **(F)** Grand average of hydropathicity (GRAVY). *Ipomoea aquatica*, Iaq; *Ipomoea batatas*, Ib; *Ipomoea cairica*, Ica; *Ipomoea nil*, Inil; *Ipomoea trifida*, Itf; *Ipomoea triloba*, Itb. **(G)** Subcellular localization of *IbGRAS37*, *IbGRAS44*, and *IbGRAS62*. The images designated as GFP, Bright, and Merged represent the green fluorescence field, bright field, and superposition field, respectively. Bars = 20 μm.

To ensure the identification of DELLA family members, HMM and BLAST methods were employed for identification. The HMM identifier for DELLA is PF02309, and the BLAST input sequences were published DELLA family members from sweet potato. After filtering, we identified 20 members of the DELLA protein family in six *Ipomoea* species (**Supplementary Table S1**). We analyzed the physicochemical properties of the proteins, revealing that most DELLA proteins have amino acid counts between 500-600, with molecular weights typically in the range of 56-65 kDa, except for smaller *InilDELLA3* and *InilDELLA5*, and notably larger *ItfGRAS38* (**Figures 1A, B**). The isoelectric point (pI) values of most proteins range from 5.0 to 5.5, indicating these genes are prefer acidity. *InilDELLA3* and *InilDELLA5* have higher pI values, indicating their alkali tolerant nature. The instability index of most proteins falls between 42-49, indicating that these proteins are unstable. (**Figures 1C, D**). The aliphatic index of DELLA proteins exhibits a range of 70.15 to 91.7, with the majority of instances falling within the interval of 75 to 90. A higher aliphatic index generally indicates greater thermal stability of the protein. *InilDELLA4* (91.7) may have higher heat tolerance than *InilDELLA3* (70.15). Similar genes from different species may have comparable aliphatic indices. For instance, *IaqDELLA3* and *IcaDELLA3* both have an aliphatic index of 90.23. The mean hydropathicity of the proteins encoded by *DELLA* genes is consistently negative, indicating an overall hydrophilic character. However, the extent of hydrophilicity varies among these proteins. The majority of these proteins display GRAVY values within the range of -0.2 to -0.3, indicating a moderate level of hydrophilicity. It can be observed that certain proteins, including *IaqDELLA3*, *IbGRAS37*, *IcaDELLA3*, *InilDELLA4*, and *ItbDELLA3*, exhibit relatively higher grand average of hydropathicity values, suggesting a lower degree of hydrophobicity compared to the other proteins (**Figures 1E, F**).

Subcellular localization predictions indicate that the majority localize to the nucleus, with some (namely, *IaqDELLA3*, *IbGRAS37*, *IcaDELLA3*, *ItfGRAS10*, and *ItbDELLA3*) localizing to both the cytoplasm and the nucleus. The results of the signal peptide predictions indicate that the majority of the proteins possess nuclear export signals (**Supplementary Table S1**). The subcellular localization of DELLA proteins in sweet potato was further investigated by expressing *DELLA*-enhanced GFP (eGFP) fusion proteins under the control of the CaMV 35S promoter in *Nicotiana benthamiana* leaf hypodermal cells. Empty eGFP vector was transfected into *N. benthamiana* leaf hypodermal cells as a control. As anticipated, eGFP was observed to be distributed uniformly throughout the cytoplasm and the nucleus, while the *DELLA* proteins of sweet potato are localized in the nucleus (**Figure 1G**).

Taken together, these observations indicate that the DELLA protein family exhibits highly conserved features across different *Ipomoea* species. Nevertheless, there are some discrepancies between DELLA families in different species, which may be indicative of functional specialization and species-specific adaptive changes.

### Consistency between *Ipomoea* species evolution and *DELLA* gene evolution

**Figure 2:**
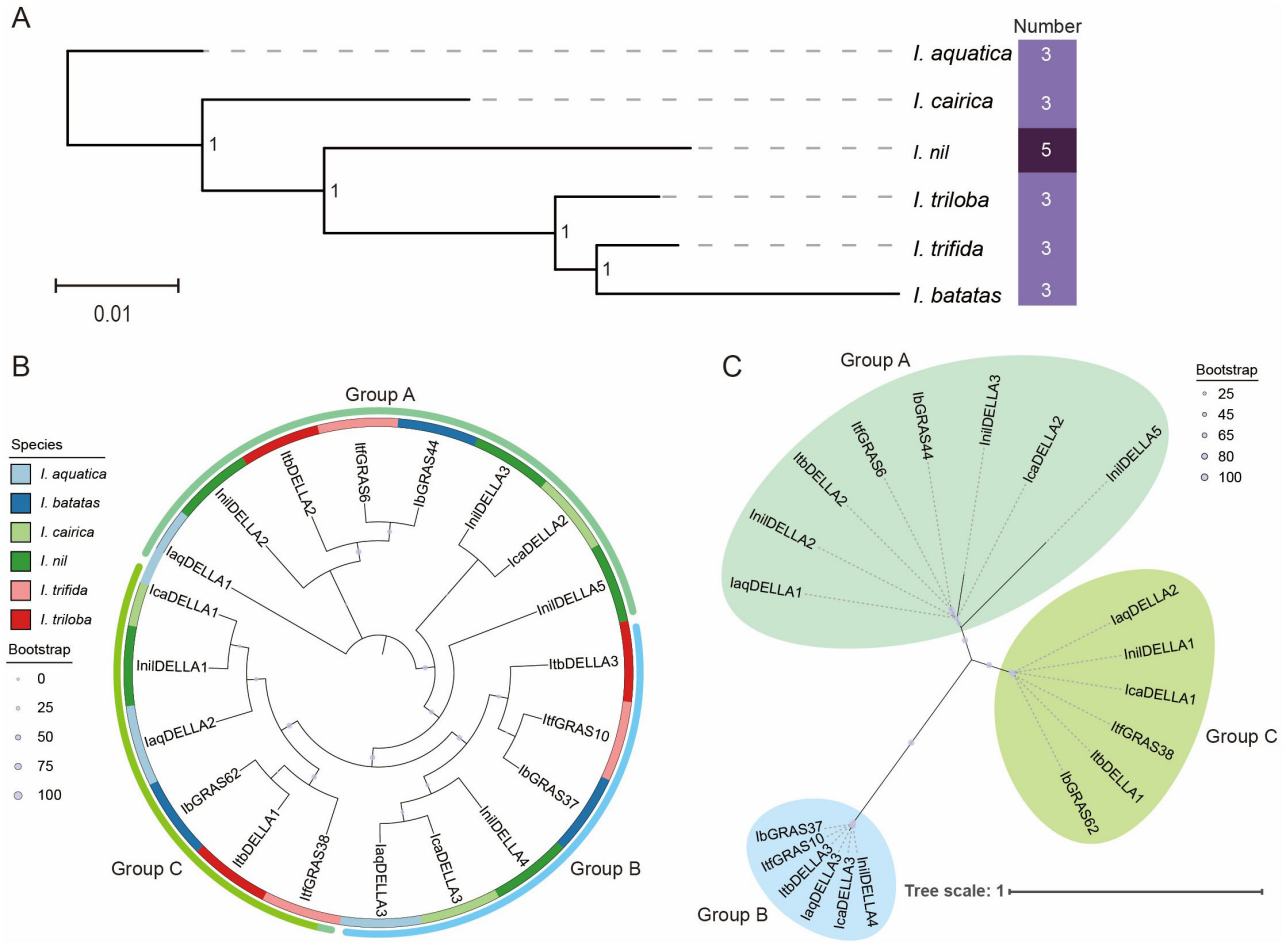
The phylogenetic tree of *DELLA* genes within these species. **(A)** The evolutionary relationships between sweet potato and five related species. Values at the nodes indicate bootstrap support (1 = 100%). The heatmap illustrates the number of *DELLA* genes across six *Ipomoea* species. **(B)** Phylogenetic tree of *DELLA* genes without evolutionary rates, reflecting the phylogenetic relationships and branching order among six *Ipomoea* species. The full-length amino acid sequences of DELLA proteins were aligned using MAFFT, and the tree was constructed using the maximum-likelihood (ML) method implemented in IQ-tree. Genes belonging to the same organism were marked with the same color. **(C)** Phylogenetic tree of *DELLA* genes with evolutionary rates, reflecting the inferred evolutionary speed of genes among six *Ipomoea* species. The length of branch represents the evolutionary distance.

Initially, we constructed a phylogenetic tree for the *six Ipomoea species*, with the aim of elucidating the evolutionary relationships within this clade. The results indicated that *I. aquatica* is located at the base of the tree, suggesting that it is the earliest diverging member among the six *Ipomoea* species, while *I. batatas* and *I. trifida* diverged relatively later. In contrast, *I. batatas* and *I. trifida* are the most closely related species, forming a sister group. The analysis of branch lengths revealed that *I. trifida* exhibits shorter branches, whereas *I. nil* displays longer branches, which indicate that *I. nil* has undergone rapid evolutionary changes. Of the six species examined, five species (*I. aquatica*, *I. batatas*, *I. cairica*, *I. trifida*, and *I. triloba*) were found to possess three *DELLA* genes, while *I. nil* was observed to possess five *DELLA* genes (**Figure 2A**). This uniformity in the number of *DELLA* genes may be indicative of the conservation of the *DELLA* gene family across *Ipomoea* species. The variation in the number of *DELLA* genes between *Ipomoea* species suggests that the *DELLA* gene family may have undergone gene duplication events in certain lineages, resulting in multiple *DELLA* genes in different species.

To gain further insight into the phylogenetic relationships among *Ipomoea* species, we compared the DELLA protein sequences to jointly construct a phylogenetic tree. The results revealed that the 20 *DELLA* genes from six *Ipomoea* species were classified into three groups. Group A comprises the largest number of *DELLA* genes among the groups, with eight genes in total. Groups B and C each comprise six *DELLA* genes. The *DELLA* genes from the same species were distributed across different groups. For example, the *DELLA* genes from *I. batatas* appeared in Groups A, B, and C. This distribution indicates that the DELLA protein family in these species may have formed through gene duplication events and acquired varying degrees of functional differentiation during evolution (**Figure 2B**). A certain degree of homology was observed among *DELLA* genes from different species based on the phylogenetic topology. For example, *DELLA* genes in Group C were found to cluster together, suggesting that they have a common ancestral origin. Additionally, DELLA proteins from closely related species (*I. batatas*, *I. triloba* and *I. trifida*) frequently clustered together (*ItfGRAS38*, *ItbDELLA1*, and *IbGRAS62*), reflecting the consistency between species evolution and gene evolution. The phylogenetic tree displays some elongated branches, such as those of *InilDELLA5* and *InilDELLA3*, which may indicate that these genes may have undergone a rapid evolutionary rate. The branch lengths of some gene pairs (*IbGRAS62* and *ItbDELLA1*) are almost zero, suggesting that they may be recently diverged homologous genes or subject to strong functional constraints (**Figure 2C**). Overall, the evolutionary history of the DELLA family in the genus *Ipomoea* is complex and potentially involves gene duplication, functional differentiation, and other evolutionary events.

### The diversity in gene structure and motif plays a role in the functional diversification of *DELLA* genes in *Ipomoea* species

**Figure 3:**
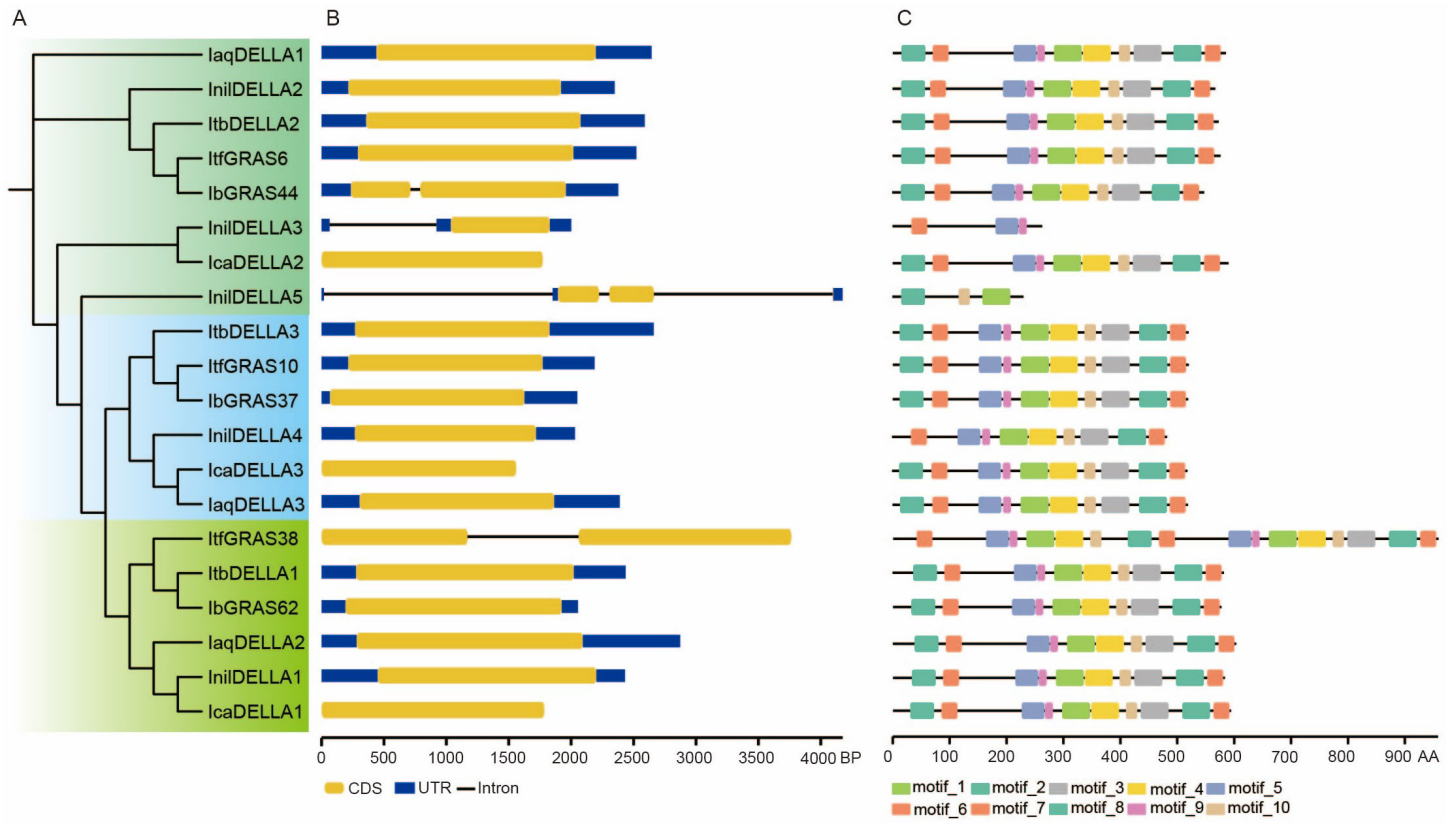
Comparison of domains, gene structure, and conserved protein motifs of *DELLA* genes in six *Ipomoea* species. **(A)** The Neighbor-Joining (NJ) phylogenetic tree was constructed based on the full-length sequences of DELLA proteins in six *Ipomoea* species. The genes are divided into three groups according to the evolutionary tree. Group A is labeled light green, group B is labeled blue and group C is labeled chartreuse. **(B)** The exon-intron structure of *DELLA* genes in six *Ipomoea* species. Yellow boxes indicate coding sequences (CDSs), blue boxes indicate untranslated regions (UTRs), and black lines represent introns. **(C)** The motif composition of *DELLA* genes in six *Ipomoea* species. The motifs, numbered from 1 to 10, are displayed in boxes of varying colors.

The gene structure, motifs, and domains are correlated with their functions. A phylogenetic analysis of the conserved DELLA and GRAS domains of 20 genes revealed that all genes contain these domains (**Supplementary Table S1**). This identify result is consistent with that of a previous study (Chen et al., 2019). The gene structure analysis indicates that the majority of *DELLA* genes possess a single CDS, while some genes own two CDSs. *InilDELLA3* and *InilDELLA5* display the insertion of introns within the 5’ untranslated region (UTR) of the gene. The *DELLA* genes within the same clade have comparable lengths and structures (**Figures 3A, B**). These observations suggest that genes with different structures may possess distinctive biological functions.

Identification of conserved motifs revealed that InilDELLA5 lacks motif 1, while InilDELLA3 and InilDELLA4 lack motif 2. Motifs 3-10 are present in the majority of *DELLA* genes. Furthermore, ItfGRAS38 displays multiple repeated motifs, which may be attributed to partial gene duplication (**Figure 3C**). In any case, the conservation of structure and motifs within subfamilies may serve to corroborate the results of the phylogenetic analysis.

### *Cis*-elements of *DELLA* gene promoters reveal complex regulatory mechanisms in the *Ipomoea* species

Regulation of gene expression is typically mediated by *cis*-acting elements located within the upstream promoter sequence. These *cis*-elements, located in the non-coding DNA upstream of the gene transcription start site, regulate the stress receptor or tissue-specific expression behavior of genes under different environmental conditions. It is therefore essential to analyze the *cis*-elements that may be involved in the regulation of *DELLA* genes, as this is a crucial step in understanding the regulatory mechanism of *DELLA* genes and evaluating their potential functions. A comprehensive analysis of the *cis*-elements in *DELLA* gene promoters was conducted based on phylogenetic analysis. The results indicate that the majority of gene promoters in Group A contain ABRE, GATA-motif, LAMP-element, and RY-element sequences, whereas these elements are less prevalent in Group B genes. This finding suggests that genes in Group A are likely to be involved in stress response (ABRE), photosynthesis regulation (GATA), and seed development (LAMP, RY). Most of the Group B gene promoters contain CARE, AuxRP-core, CCAAT-box, and LTR elements, whereas these elements are less common in Group A and C gene promoters. This suggests that genes in Group B are primarily involved in growth regulation (AuxRP-core), signal transduction (CARE), and environmental response (LTR). Most gene promoters in Group C contain A-box, CCGTCC-box, and TGA-box elements, whereas these elements are less prevalent in Group A and B gene promoters. This finding suggests that genes in Group C are primarily involved in tissue-specific expression (A-box), cell cycle regulation (CCGTCC-box), and plant defense responses (TGA-box). These results highlight the functional diversity of the *DELLA* genes. Such differences may be due to evolutionary divergence between species and the need to adapt to different environmental conditions.

**Figure 4:**
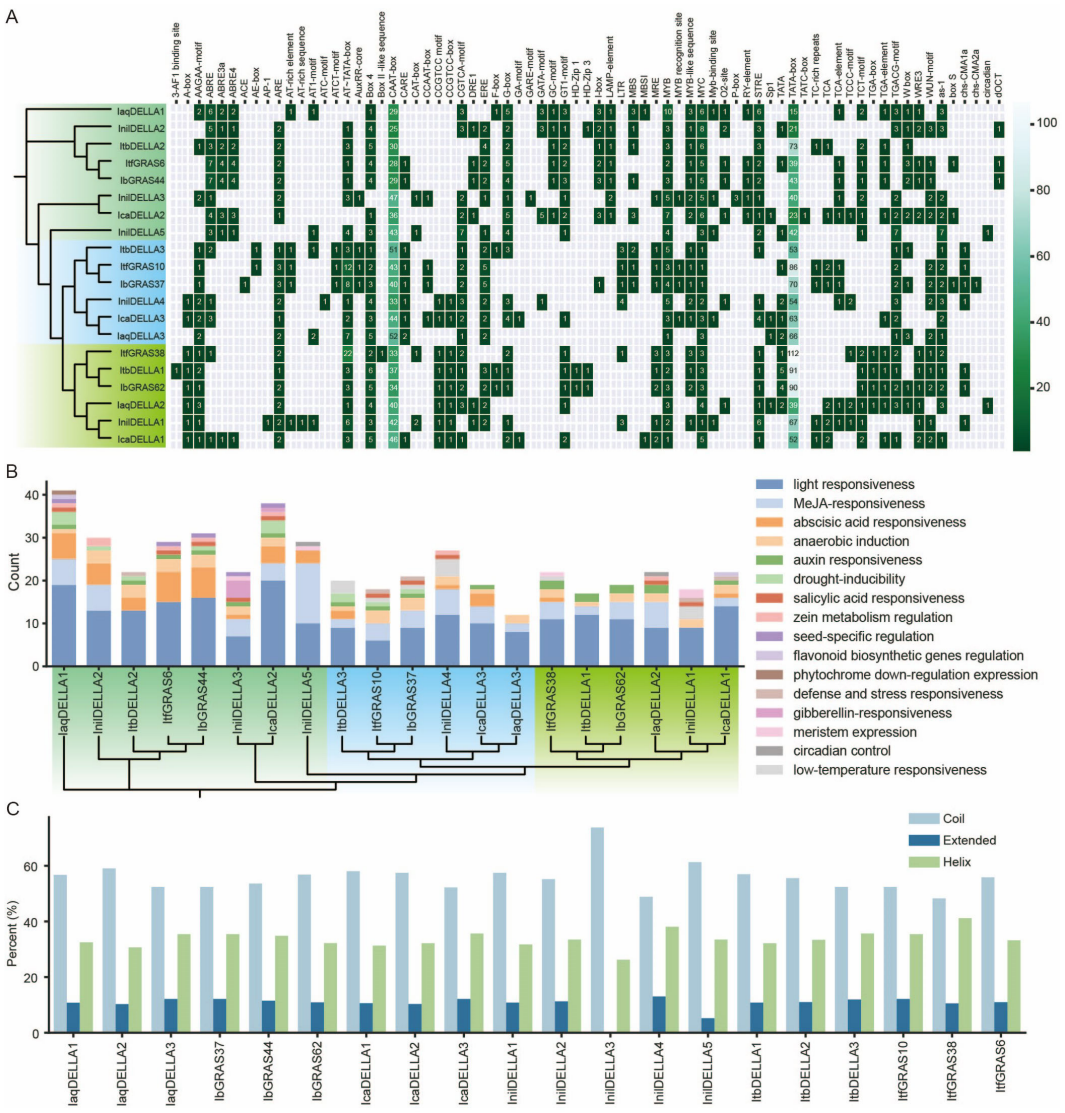
Analysis of *DELLA* gene promoters. **(A)** *Cis*-elements in the promoters of the *DELLA* genes in six *Ipomoea* species. The genes are divided into three groups according to the evolutionary tree. Group A is labeled light green, group B is labeled blue and group C is labeled chartreuse. **(B)** The number of functional *cis*-elements present in the promoters of the *DELLA* genes across six *Ipomoea* species. **(C)** 3-D structure analysis of the DELLA protein.

Additionally, our results suggest that *IaqDELLA1* is evolutionarily distant from other genes, forming an independent branch. The *cis*-acting element composition of IaqDELLA1 is significantly different from that of other genes, with a reduced number of CAAT-boxes (29) and TATA-boxes (15). This suggests that *IaqDELLA1* may have distinct transcriptional regulation and functional properties compared to other genes. *IcaDELLA2* and *InilDELLA3* are found to cluster together and have similar compositions of *cis*-acting elements, with comparable numbers of ABRE (4 and 1), CAAT-box (36 and 47) and TATA-box (23 and 40). This indicates the possibility of analogous patterns of expression regulation. The *IaqDELLA3*, *IcaDELLA3*, and *InilDELLA4* genes form a large cluster with the *IbGRAS37*, *ItfGRAS10*, and *ItbDELLA3* genes. The high content of AT-rich elements observed in *IbGRAS37* and *ItfGRAS10*, in comparison to the almost complete absence of such elements in *IaqDELLA3*, *IcaDELLA3*, and *InilDELLA4*, suggests the loss and gain of regulatory elements between the sub-branches. *InilDELLA2* clusters with *IbGRAS44*, *ItfGRAS6* and *ItbDELLA2*, yet it forms a discrete sub-branch. The ABRE content of the four genes is similar, but *InilDELLA2* has a slightly lower TATA-box than the other three genes, indicating some differences in the composition of regulatory elements. Additionally, *InilDELLA5* and *IaqDELLA2* contain a circadian element, whereas the remaining genes lack this feature, indicating the potential for a distinctive circadian rhythm expression (**Figure 4A**). Subsequently, the number of elements present in specific biological pathways was counted. The results demonstrate that light responsiveness and methyl jasmonate (MeJA) responsiveness are the most prevalent regulatory characteristics. Abscisic acid (ABA) responsiveness is observed in many genes, with the quantity exhibiting considerable variation. The regulatory features of seed-specific regulation and zein metabolism regulation are present in only a few genes (*IaqDELLA1*, *ItfGRAS6*, *IbGRAS44*, *InilDELLA3*, *IcaDELLA2*, *InilDELLA2*, *InilDELLA4*, and *IaqDELLA2*) (**Figure 4B**).

### The secondary and 3-D structures of DELLA proteins in *Ipomoea* species display notable variations

To further elucidate the structural characteristics of the DELLA protein in six *Ipomoea* species, we analyzed the secondary structure of the proteins. The results revealed that in all proteins, the proportion of ‘Coil’ exceeded 50%, indicating that it was the predominant secondary structure. The proportion of extended structures was generally low, around 10-12%. The proportion of helix structures was between coil and extended, approximately 30-35% (**Figure 4C**). Notable differences were observed in the secondary structure proportions of the DELLA protein across different species. For example, IaqDELLA1 and InilDELLA5 had almost identical helix proportions, but their extended proportions showed significant differences.

Additionally, AlphaFold3 was used to predict the 3-D structure of DELLA proteins. The results showed both similarities and differences in the 3-D structures of various DELLA proteins. For instance, the majority of DELLA proteins featured a prominent loop following the conserved DELLA domain. Some genes exhibited the presence of helices preceding the DELLA domain, whereas in the case of IaqDELLA1 and InilDELLA3, these structures were absent (**Supplementary Figure S1**). Overall, significant differences were also observed in the secondary structure proportions of different genes within the same species. For example, the coil proportions of InilDELLA1 and InilDELLA3 differed significantly. The secondary structure proportions of the same gene may vary between different species, possibly due to evolutionary divergence. Furthermore, variations in secondary structure proportions were observed between different genes within the same species, probably due to functional differences.

### Multiple positional rearrangements of *DELLA* genes occurred in *Ipomoea* species

To investigate the evolutionary dynamics of the *DELLA* genes, a synteny analysis was performed based on the evolutionary relationships of six *Ipomoea* species. The results revealed that *IaqDELLA1*, *IaqDELLA2*, and *IaqDELLA3*, which are situated at the base of the *Ipomoea* species evolution, have syntenic relationships with other species. The selected genus *Ipomoea* contains three groups of syntenic *DELLA* gene pairs. The first group (Group s1) contains the following syntenic *DELLA* gene pairs: *IaqDELLA1*-*IcaDELLA2*-*InilDELLA2*-*ItbDELLA2*-*ItfGRAS6*-*IbGRAS44*. The second group (Group s2) consists of the following pairs: *IaqDELLA3-IcaDELLA3-InilDELLA4-ItbDELLA3-ItfGRAS10-IbGRAS37*. The third group (Group s3) is made up of the following pairs: *IaqDELLA2*-*IcaDELLA1*-*InilDELLA1*-*ItbDELLA1*-*ItfGRAS38*-*IbGRAS62*.

Furthermore, the syntenic *DELLA* genes in the six *Ipomoea* species are consistently located on two chromosomes per species, suggesting that they have remained conserved throughout the evolutionary process. Rearrangements in the order of the syntenic genes were identified between the s1 and s2 groups. For example, *IaqDELLA1* and *IcaDELLA2* have a syntenic relationship, as do *IaqDELLA3* and *IcaDELLA3*. In *I. aquatica*, the order of the syntenic *DELLA* genes on the same chromosome is *IaqDELLA1*-*IaqDELLA3*. *I. cairica*, however, the chromosomal order is altered, with *IcaDELLA3*-*IcaDELLA2* occurring instead.

**Figure 5:**
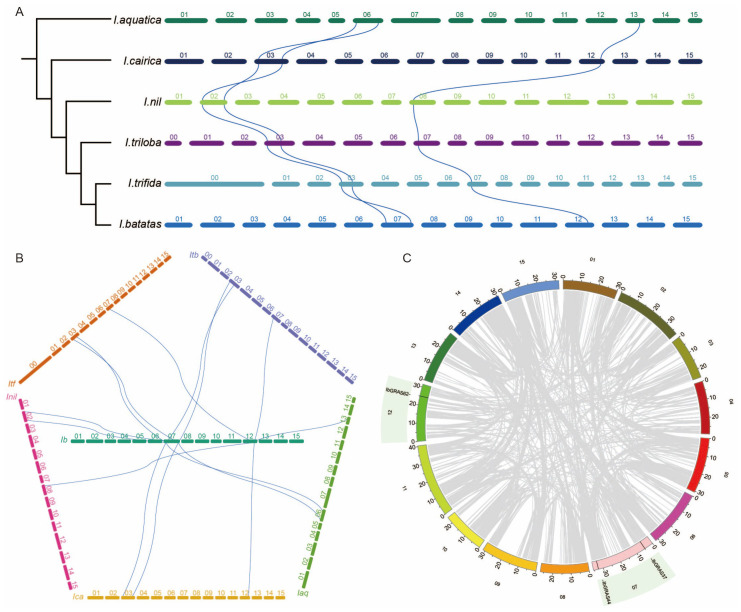
Synteny analysis of the *DELLA* genes. **(A)** Synteny analysis based on the phylogeny of six *Ipomoea* species. The phylogeny of the six *Ipomoea* species indicates the evolutionary order. The blue lines represent syntenic *DELLA* genes. **(B)** Synteny analysis of *DELLA* genes between sweet potato and the other five *Ipomoea* species. The blue lines represent syntenic *DELLA* genes. **(C)** Synteny analysis and the positions of *DELLA* genes in sweet potato. Grey lines indicate other syntenic genes.

Subsequently, a series of positional rearrangements occurred. In *I. nil*, *I. triloba*, and *I. trifida*, the syntenic *DELLA* genes did not undergo rearrangements, indicating evolutionary stability (**Figure 5A**). Then, a synteny analysis was performed between *I. batatas* and the other five *Ipomoea* species. The results demonstrated that the three *DELLA* genes in *I. batatas* have syntenic relationships with the *DELLA* genes in the other five species, indicating a high conservation of the *DELLA* genes in *I. batatas* (**Figure 5B**). An intraspecies synteny analysis of *I. batatas* revealed the absence of syntenic relationships among *DELLA* genes within the *I. batatas* genome, suggesting functional divergence of *DELLA* genes within this species (**Figure 5C**). The Duplicate_gene_classifier tool in MCScanX was employed to determine the duplication modes of *DELLA* genes in *Ipomoea* species. The primary duplication mode of *DELLA* genes in *Ipomoea* species was identified as dispersed duplication (**Supplementary Table S1**).

### *DELLA* genes response to abiotic stresses in sweet potato

To explore the potential functions of the *DELLA* genes, we analyzed transcriptome data from sweet potato referring to tissue expression, hormonal response and abiotic stresses. The results indicated that the expression levels of the three genes were significantly higher in the aerial parts of the Xuzi3 variety than in the underground parts. The highest expression levels of *IbGRAS62* were observed in all tissues of Xuzi3, while the lowest expression levels were observed for *IbGRAS37*. In the Yan252 variety, the expression of *IbGRAS37* in the aerial parts was observed to be lower than that observed in the underground parts. Conversely, the expression of *IbGRAS44* and I*bGRAS62* in the aerial parts was found to be higher than that observed in the underground parts. Of the three genes, *IbGRAS62* exhibited the highest expression levels in all tissues. These indicated that group s1 may mainly function in the aerial parts, group s2 may function mainly in the underground parts, and group s3 may function mainly in the whole plant. With regard to root development, the expression of *IbGRAS44* exhibited an increasing trend in the Xuzi3 variety, but a decreasing trend in the Yan252 variety. The expression of *IbGRAS62* was observed to a decline during root development in the Xuzi3 variety, whereas it exhibited an increase in the Yan252 variety.

**Figure 6:**
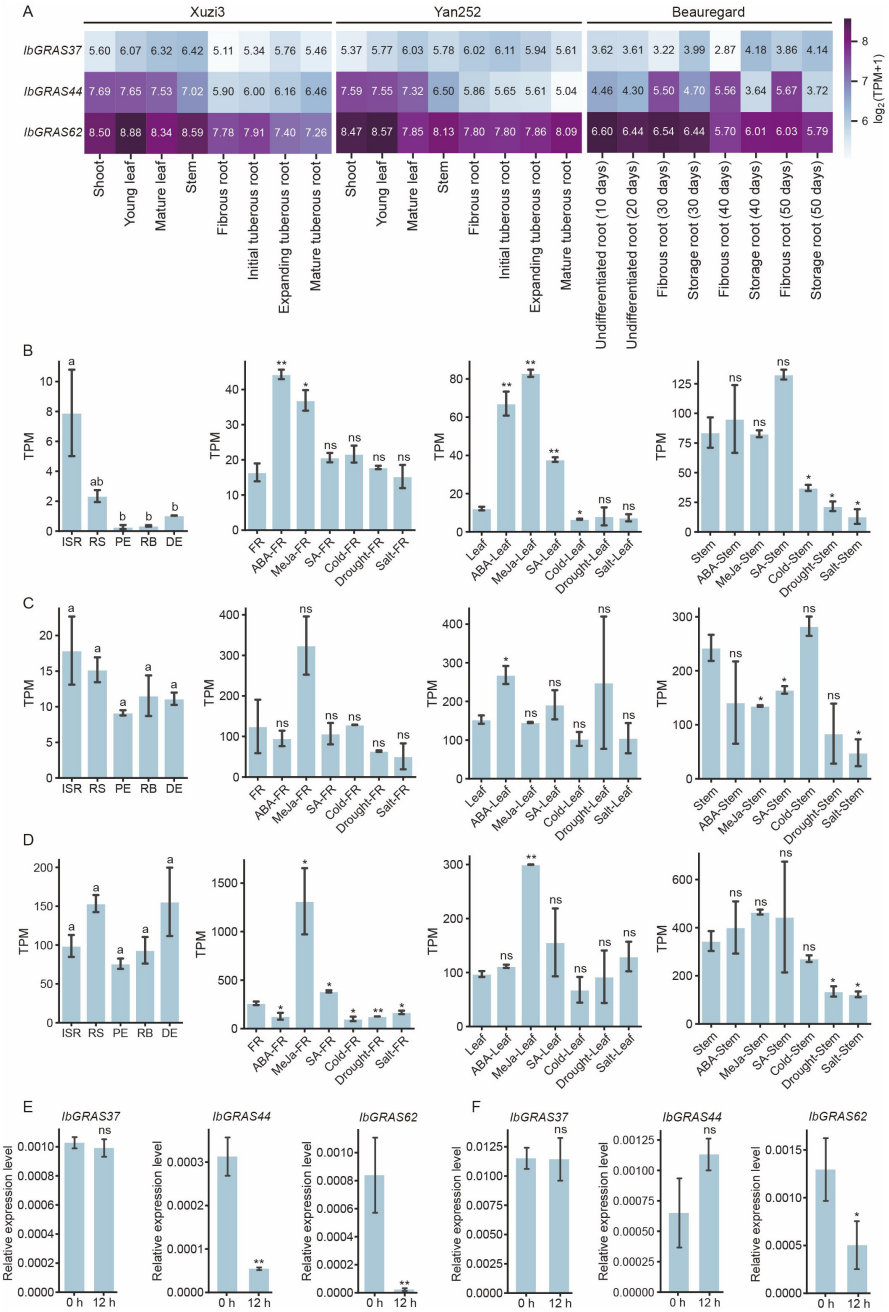
The expression pattern of the *DELLA* genes in sweet potato. **(A)** The expression pattern of sweet potato DELLA genes in different tissues across various sweet potato varieties (Xuzi3, Yan252, and Beauregard). **(B)** Expression analysis of the *IbGRAS37* gene in different parts of the roots, including hormone response and abiotic stress response in sweet potato. Various root tissues include initiative storage roots (ISR), root stalks (RS), proximal ends (PE), root bodies (RB), distal ends (DE) and fibrous roots (FR). **(C)** Expression analysis of the *IbGRAS44* gene in different parts of roots, including hormone response and abiotic stress response in sweet potato. **(D)** Expression analysis of *IbGRAS62* gene in different parts of the roots, including hormone response and abiotic stress response in sweet potato. **(E)** Relative expression levels of *IbGRAS37*, *IbGRAS44* and *IbGRAS62* in sweet potato seedlings after 12 hours of ABA treatment. **(F)** Relative expression levels of *IbGRAS37*, *IbGRAS44* and *IbGRAS62* in sweet potato seedlings after 12 hours of 20% PEG treatments. Different letters represent a significant difference at *P* <0.05 determined by least significant difference (LSD) test. A two-tailed Student’s *t*-test was used to determine *P* values, *P < 0.05, **P<0.01; ns, no significant difference.

To further clarify the effect of the *DELLA* gene on sweet potato root development, an analysis of the root development transcriptome of the Beauregard variety was conducted. The results showed that the expression of the three genes was relatively stable in undifferentiated roots, with *IbGRAS62* showing the highest expression levels and *IbGRAS37* displaying the lowest expression. In fibrous and storage roots at 30, 40, and 50 days, it was observed that *IbGRAS37* expression was higher in fibrous roots than in storage roots, while *IbGRAS44* expression was higher in storage roots than in fibrous roots. The expression of *IbGRAS62* was relatively balanced in both fibrous and storage roots at these stages, indicating that *IbGRAS37* may function primarily in fibrous roots, whereas *IbGRAS44* may function primarily in storage roots (**Figure 6A**).

To gain further insight into their expression in different root tissues, we analyzed transcriptome data from the Xu18 variety, comprising different root tissues, including initiative storage roots (ISR), root stalks (RS), proximal ends (PE), root bodies (RB), distal ends (DE) and fibrous roots (FR). Expression of the three genes (*IbGRAS37*, *IbGRAS44*, and *IbGRAS62*) in different parts of the sweet potato root showed that they were expressed in various parts of the sweet potato but with notable differences in expression levels. The overall expression level of *IbGRAS37* was found to be lower than that of *IbGRAS44* and *IbGRAS62*, with relatively higher levels observed in FR and relatively lower levels in PE, RB, and DE. The expression level of *IbGRAS44* was observed to be higher than that of *IbGRAS37*. The highest expression was observed in FR, followed by ISR and RS, and relatively low in PE, RB, and DE. The expression level of *IbGRAS62* was significantly higher than the other two genes in all parts examined, especially in ISR, RS, RB, DE, and FR. Overall, the high expression of *IbGRAS62* in ISR, RS, RB and DE suggests that it may play a key role in the formation and development of storage roots. *IbGRAS44* may also be involved in this process. Conversely, *IbGRAS37* may play a role primarily in the leaves (**Figures 6B-D**).

To gain further insight into the function of *DELLA*, an analysis of the hormone response and stress transcriptome of the Xu18 variety was performed. The results showed that *IbGRAS37* was induced and upregulated by ABA, MeJa, and salicylic acid (SA) in the leaves. In the stems, the expression of this gene was found to be upregulated by SA and downregulated under cold, drought, and salt stress conditions. In FR, its expression was found to be upregulated by ABA and MeJa. *IbGRAS44* was upregulated by ABA and downregulated under cold and salt stress in the leaves. In the stems, it was downregulated by MeJa and SA, as well as suppressed under drought and salt stress conditions. In FR, its expression was upregulated by MeJa and downregulated under drought and salt stress. *IbGRAS62* was upregulated by MeJa in both the leaves and the stems. Its expression was downregulated under cold, drought, and salt stress in the stem. In FR, the gene was observed to be upregulated by MeJa and SA, and conversely, downregulated under cold, drought, and salt stress.

Subsequently, sweet potato seedlings were treated with ABA and 20% PEG for a period of 12 h. Compared to the initial time point (0 h), the expression levels of *IbGRAS44* and *IbGRAS62* were reduced at 12 h under ABA treatment (**Figure 6E**). Under 20% PEG treatment, the expression of *IbGRAS44* increased at 12 h, while *IbGRAS62* showed decreased expression at the same time point (**Figure 6F**). Overall, the response patterns of the three genes to hormones and stress in different tissues (leaves, stems, and fibrous roots) exhibited variability, suggesting that they may have tissue-specific functions.

### *DELLA* genes and other genes synergistically regulate the growth and development of sweet potato

**Figure 7:**
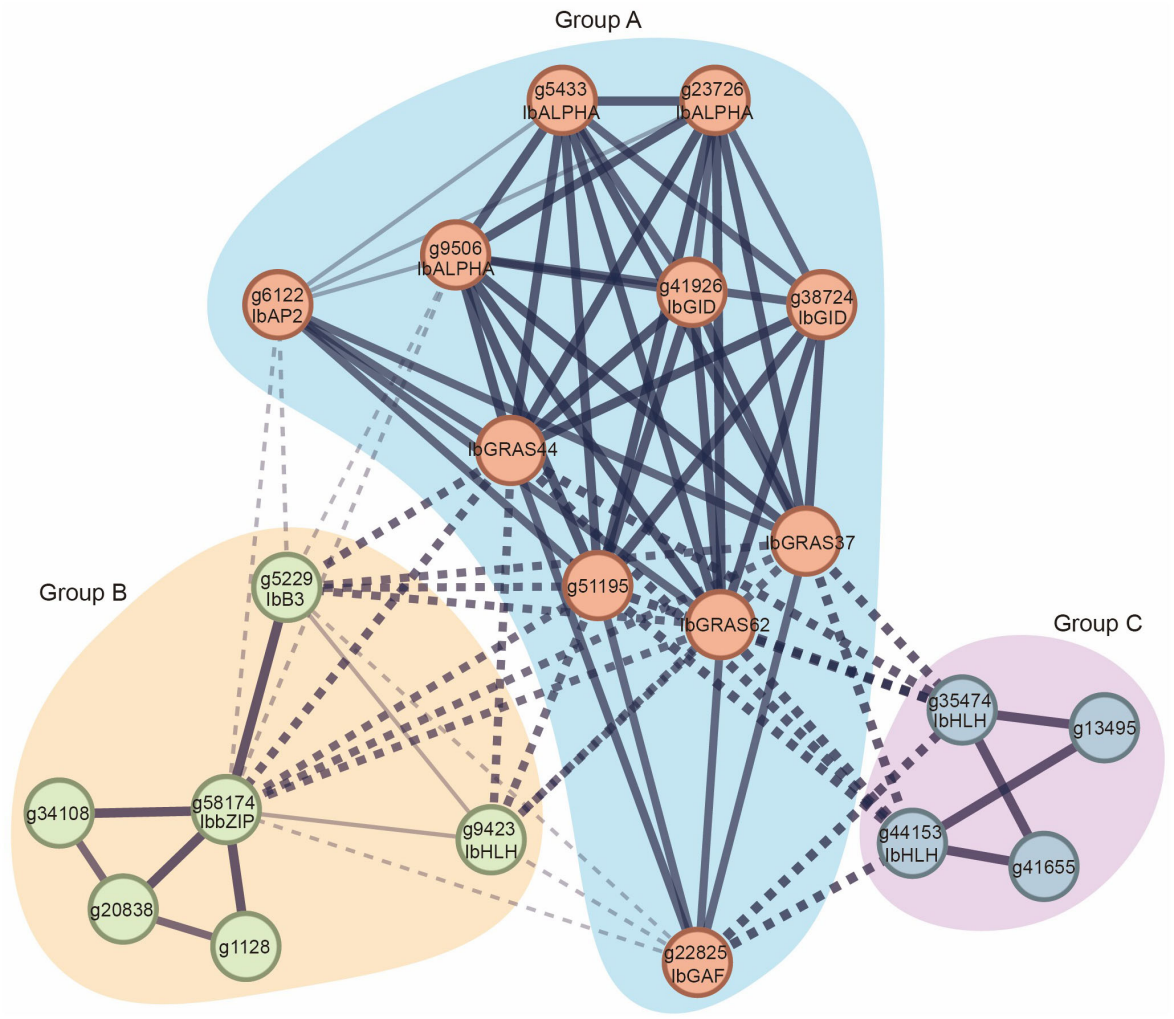
Interaction network of DELLA proteins in sweet potato. The MCL clustering method was used for analysis. The thickness of the solid black lines represents the strength of interactions between genes, while the type of the line indicates interactions among different groups.

To gain a more profound understanding of the functions and potential mechanisms of the sweet potato DELLA protein, we employed the String database to construct a comprehensive sweet potato genome interaction network. By clustering based on genes, a total of 7,569 interacting clusters were identified in sweet potato (**Supplementary Figure S2**). Subsequently, the interaction relationships of the DELLA proteins were extracted. In accordance with the aforementioned interactions, the sweet potato DELLA proteins were categorized into three groups: Group A genes are capable of interacting with genes in Groups B and C, whereas genes in Groups B and C are unable to interact with each other. To elucidate the function of DELLA, we identified the conserved domains contained in the DELLA direct interaction genes. Our results indicate that the three sweet potato DELLA proteins interact with proteins in Group A (g22825, g6122, g9506, g41926, g38724, g5433, and g23726), which predominantly contain GAF, AP2, ALPHA, and GID conserved domains. Additionally, the three DELLA proteins were found to interact with proteins in Group B (g5229, g58174, and g9423), which contain B3, bZIP, and HLH domains. They also interact with proteins in Group C (g35474 and g44153), which contain HLH domains (**Figure 7**). In other species, it has been demonstrated that DELLA proteins interact with proteins containing these domains, thereby regulating a number of important processes, including stem elongation, seed germination, flowering time regulation, leaf and root development, stress resistance, and plant hormone signal transduction (Davière and Achard, 2013; Olszewski et al., 2002; Peng and Harberd, 2002; Richards et al., 2001; Sun and Gubler, 2004).

## Discussion

### Evolutionary conservation and diversification of *DELLA* genes in *Ipomoea* species

The DELLA family was found to show both conservation and remarkable diversification within the six *Ipomoea* species. The species *I. nil* has the highest number of *DELLA* genes, which may indicate that this species has more complex regulatory mechanisms in DELLA-mediated signaling pathways. It is possible that certain *DELLA* genes (such as *InilDELLA5*) may have undergone rapid evolution or functional differentiation, resulting in significant sequence divergence from other *DELLA* genes. The variation in the number of *DELLA* genes observed between different species may reflect their different requirements in response to environmental stresses or in the regulation of growth and development.

The physicochemical properties and the secondary and tertiary structures of the proteins encoded by the *DELLA* genes, exhibit significant differences among the six *Ipomoea* species. DELLA proteins regulate plant growth and development through the degradation triggered by the GA signaling pathway. Most DELLA proteins have a high instability index, indicating that they are likely to be short-lived. This instability provides plants with a flexible mechanism for responding to environmental signals, enabling rapid adjustment of growth rates. DELLA proteins, which have a moderate molecular weight (50-65 kDa), can interact with multiple proteins due to their flexible structure, in particular with other components of the GA signaling pathway (Arkin et al., 2003). The high hydrophilicity of DELLA proteins supports their functional activity in the aqueous environment of the nucleus, particularly during transcriptional regulation, facilitating interactions with DNA, transcription factors, and signaling complexes (Nelson and Steber, 2016). Most DELLA proteins have a low isoelectric point, indicating that they are negatively charged. Negatively charged DELLA proteins are more likely to bind to positively charged transcription factors or DNA, helping to form of complexes that repress GA-responsive gene expression in the nucleus (Norel et al., 2001). The high aliphatic index of DELLA proteins ensures their functional stability across different growth temperatures, allowing for sustained activity under different environmental conditions. Additionally, the subcellular localization information of DELLA proteins further reveals their multifunctionality. The nuclear localization and nuclear export signals of DELLA proteins are closely linked to their dynamic nucleocytoplasmic transport mechanisms. Regulation of DELLA protein distribution within and between the nucleus enables plants to respond rapidly to environmental changes or hormonal signals, thereby controlling growth and development (Chinnusamy et al., 2008). Additionally, the DELLA proteins IbGRAS44 and IbGRAS37 in sweet potato exhibit a high aliphatic index and low GRAVY values, which may contribute to enhanced stability and efficiency under environmental stresses such as drought or salt stress. The physicochemical properties of DELLA proteins may confer increased stress tolerance in sweet potato, thereby improving the plant’s resilience and survivability. The remarkable differences and variability indicate a considerable level of complexity, which may be linked to gene duplication events or variations in genome size (Fan et al., 2021). The presence of introns can significantly influence both gene transcription and the splicing processes. Most *DELLA* genes are devoid of introns, suggesting that this gene family has a relatively high efficiency of gene expression and maintains strict regulatory mechanisms. Furthermore, several studies have demonstrated that more ancient gene families generally possess fewer introns (Fedorova and Fedorov, 2003). This phenomenon can be attributed to the prolonged periods of selection and optimization that these gene families have undergone during evolution, which has resulted in a reduction in their structural complexity (Lynch and Richardson, 2002). Some *DELLA* genes possess introns and multiple exons, indicating that these genes may be subject to more complex post-transcriptional regulation. Besides, different genes show diversity in their overall structure and motif combinations. This diversity may confer distinct functional capabilities on these genes, enabling their involvement in a range of biological processes. The arrangement and combination of motifs in different genes vary, which may result in subtle functional differences in the proteins they encode. This allows the proteins to fulfil different roles in a variety of biological processes and in response to different environmental conditions.

Analysis of *cis*-acting elements in *DELLA* gene promoters reveals both conservation and differences between species. Orthologous gene promoters across the six species (such as *DELLA* genes in different species) often retain common *cis*-element characteristics, which reflect functional conservation. Simultaneously, orthologous gene promoters in different species also exhibit differences in *cis*-element composition, which indicate the evolution of species-specific regulatory mechanisms. The presence of MYB and MYC binding sites are distributed in the majority of gene promoters, suggesting that these transcription factors may exert a substantial influence on the regulatory network of GRAS family genes (Waseem et al., 2022). Certain gene promoters (such as *IaqDELLA1* and *InilDELLA3*) are enriched for specific transcription factor binding sites, which may indicate more sophisticated transcriptional regulation. Additionally, *DELLA* genes may exhibit spatio-temporal specificity in expression. For example, gene promoters containing circadian elements (such as *IaqDELLA2* and *InilDELLA5*) may exhibit diurnal expression patterns. Genes containing tissue-specific elements (such as CAT-box) may exhibit elevated expression levels in specific tissues (Lin et al., 2022). The evolution of *cis*-elements may be synchronous with the evolution of gene sequences, as the topology of the phylogenetic tree roughly corresponds to variations in *cis*-element patterns. Meanwhile, specific clades (such as *IbGRAS62*, *ItbDELLA1*, and *ItfGRAS38*) exhibit rapid evolution of *cis*-elements, potentially reflecting novel functional specialization. Some genes, such as *IaqDELLA1* and *InilDELLA2*, have a relatively limited number of *cis*-elements in their promoters, which may indicate a more straightforward regulatory mechanism for their expression. In contrast, genes such as *IcaDELLA2* and *ItbDELLA1* contain a greater variety of *cis*-elements in their promoters, which may indicate that they are subject to more complex transcriptional regulation. These differences reflect the varying complexity of *DELLA* gene regulation among *Ipomoea* species. In general, genes that are clustered together tend to have similar compositions of *cis-*elements. However, differences in the number of individual elements may be related to their unique functions (Adjibolosoo et al., 2024; Wittkopp and Kalay, 2012). Conversely, the composition of *cis*-elements differs more significantly between major evolutionary branches, indicating a greater degree of distinct differentiation in transcriptional regulation and biological functions. The uneven distribution of *cis*-elements across evolutionary branches suggests that they may play a pivotal role in the evolution of gene expression regulation.

The *DELLA* genes in *Ipomoea* species show remarkable collinearity, which may indicate that these genes have analogous functions and regulatory expression mechanisms across these species. This positional conservation provides further evidence of the importance of these genes, as their relatively stable genomic locations help to preserve their functional integrity and efficacy (Fan et al., 2021). Additionally, the *DELLA* genes have undergone rearrangements in *Ipomoea* species, possibly due to structural genomic changes during evolution, including chromosome breakage and recombination. These changes may result in the migration of gene positions while maintaining the collinear relationships between these genes (De Storme and Mason, 2014). The collinear relationships observed indicate that, despite the gene rearrangements and structural changes that have occurred in the genes of different *Ipomoea* species during evolution, the *DELLA* genes have remained relatively conserved. This conservation may be due to the crucial roles these genes play in plant growth and development.

### Distinct roles of *DELLA* genes in sweet potato

A comparative analysis of the potential mechanisms of action of the genes *IbGRAS37*, *IbGRAS44*, and *IbGRAS62* reveals that they play distinct roles at different stages and aspects of root development. *IbGRAS37* is predominantly upregulated during the later stages of storage root development and may regulate genes related to cell wall remodeling, cell expansion, and sugar metabolism. It is likely to be involved in hormone signaling pathways, thereby promoting the expansion and storage functions of the roots (Dong et al., 2019; Kondhare et al., 2021). *IbGRAS44* displays elevated expression levels during the initial stages of fibrous root development, suggesting a role in regulating root apical meristem activity and the initial roots formation. It may also play a role in the root response to environmental stimuli and is likely to be involved in hormone signaling that promotes fibrous root branching and initial development. *IbGRAS62* is highly expressed in most tissues, particularly during the various developmental stages of both fibrous and storage roots. It is likely to be involved in energy metabolism, biosynthetic pathways, cross-regulation of multiple hormone signaling pathways, cell cycle regulation, starch synthesis, sugar transport, cell wall synthesis or modification, and the regulation of antioxidant enzyme expression, thus performing a broad and critical function throughout root development (Villordon et al., 2009; Zierer et al., 2021). Collectively, these three genes form a complex regulatory network that is involved in a number of key processes, including root morphogenesis, the development of storage functions, and environmental response. This reflects the intricate regulatory mechanisms that underpin plant root development.

The expression of *IbGRAS37* is significantly upregulated in leaves and fibrous roots under ABA and MeJa treatments, indicating that this gene may play an important role in the ABA and MeJa signaling pathways. ABA is primarily involved in responses to drought, salt, and dehydration stresses, while MeJa is typically associated with responses to biotic stresses and some abiotic stresses (Ku et al., 2018). The expression level of this gene is relatively low under drought, salt, and cold stress conditions, suggesting that it may not directly participate in these stress responses but instead functions through hormone-mediated stress signaling regulation. *IbGRAS44* is upregulated in fibrous roots following MeJa treatment and in leaves after ABA treatment, indicating that the regulation of this gene may be complex, involving multiple signaling pathways with differential hormone responses in distinct tissues. The expression of *IbGRAS44* is also significantly upregulated under cold stress, particularly in stems, suggesting that this gene may be involved in regulating physiological responses to cold stress, such as modulating membrane lipid composition and the expression of protective proteins (Wu et al., 2022). *IbGRAS62* exhibits extremely high expression under MeJa treatment, suggesting that this gene may be a key regulator in MeJa-mediated defense responses. *IbGRAS62* is likely to regulate plant responses to pathogens and mechanical damage, such as cell wall reinforcement, activation of antioxidant enzymes, and the synthesis of defense-related compounds (Appu et al., 2021). Additionally, *IbGRAS62* shows some degree of responsiveness to ABA and SA treatments in leaves, indicating that it may enhance plant tolerance to these stress conditions by interacting with jasmonic acid signaling and other stress-related pathways.

The effects of cold, drought, and salt treatments on gene expression show remarkable variability. The expression level of *IbGRAS37* in the roots exhibited a slight increase under cold stress, yet no significant alterations were observed under drought or salt stress, suggesting that this gene may be involved in adaptive root growth under cold stress, potentially regulating ion balance, osmotic adjustment, and membrane lipid composition (Zhao et al., 2021). In addition, cold treatment resulted in a reduction of *IbGRAS37* expression in the leaves and stems, indicating that cold stress may inhibit the function of this gene in these tissues. The expression of *IbGRAS44* increased in the leaves under drought stress, indicating that this gene may be closely related to drought resistance mechanisms in leaves, and may be involved in stomatal regulation, photosynthesis, and water balance (Bhargava and Sawant, 2013). Although *IbGRAS44* expression also increased in the stem under cold stress, the magnitude of this change was less pronounced than that observed under drought stress, implying that the gene may have a limited contribution to the cold response, possibly through mechanisms related to water transport, mechanical support, or the regulation of secondary growth in the stem (Gall et al., 2015). The expression of *IbGRAS62* increased in the leaves under salt stress, indicating that this gene is involved in the regulation of salt resistance in leaves. In the roots and stems, its expression decreased under cold, drought, and salt stress, suggesting that these stresses may inhibit the function of this gene in these tissues.

Although these three genes are involved in hormonal and stress responses in sweet potato, they exhibit distinct response patterns, tissue specificity, and potential functions. They are likely to represent different components of the plant’s complex stress response network, which together form a multi-layered defense mechanism that reflects the finely tuned regulatory strategies that plants have developed throughout evolution to cope with environmental change.

### Potential implications in molecular breeding

A comparative analysis of the structural, promoter element, and evolutionary characteristics of the *DELLA* genes in several species of the genus *Ipomoea* revealed the conservation, regulatory differences, and functional divergence of these genes. It is reasonable to assume that genes which are conserved across species are likely to have an essential biological function, such as the regulation of plant growth, development and stress responses. In contrast, genes that are functionally divergent may confer species-specific adaptive traits. For instance, the functional divergence of *DELLA* genes between sweet potato and its wild relatives (*I. trifida* and *I. triloba*) can provide genetic resources for sweet potato improvement, thereby aiding in the identification of functional genes with potential utility. Additionally, *DELLA* genes play a crucial role in plant responses to abiotic stresses, including drought, salinity, and cold. By analyzing the evolution of *DELLA* genes in sweet potato and its relatives, researchers can investigate how these genes help plants adapt to various environmental conditions, thus providing specific gene targets for genetic improvement of stress resistance traits. Regulating these key genes could lead to the development of sweet potato varieties with enhanced stress tolerance and improved adaptability in harsh environments. In summary, the evolutionary divergence and functional differentiation of *DELLA* genes across *Ipomoea* species provide valuable genetic resources for sweet potato improvement. It is possible that in the future, interspecies gene integration may facilitate the introduction of beneficial genes from wild relatives, thereby increasing the genetic diversity of sweet potato. This approach may assist in addressing the decline in genetic diversity in cultivated sweet potato over long breeding periods, while improving resistance to pests, diseases, and environmental stresses. This strategy has the potential for broad application potential in areas such as stress resistance, storage root development, and molecular breeding.

The complex regulatory network of DELLA proteins in sweet potato illustrates that genes in Group A are able to interact with those in Groups B and C, whereas genes within Groups B and C exhibit a lack of interaction with each other. This distinct interaction pattern implies that DELLA proteins potentially may act as key regulators in a range of physiological processes. An analysis of the conserved domains (such as GAF, AP2, ALPHA, GID, B3, bZIP, and HLH) within genes that directly interact with DELLA proteins suggests that DELLA proteins are involved in a variety of essential physiological processes. These processes include stem elongation, seed germination, flowering time regulation, leaf development, root development, stress tolerance, and plant hormone signal transduction (Du et al., 2017; Feng et al., 2020; Fukazawa et al., 2021; Galvão et al., 2012; de la Rosa et al., 2014; Pullen et al., 2019; Rombolá-Caldentey et al., 2014; Vera-Sirera et al., 2016). These results not only confirm the established functions of DELLA proteins observed in other species, but also potentially reveal specific functions of DELLA proteins unique to sweet potato.

## Conclusion

This study presents a comprehensive genomic analysis of the *DELLA* gene family members in six *Ipomoea* species. The results showed that six *Ipomoea* species possess 20 members of the *DELLA* gene family. A more detailed analysis of the distribution, organizations and structure of the *DELLA* gene family within the genus *Ipomoea* suggests that these genes have undergone complex evolutionary processes. It is noteworthy that the syntenic analysis revealed that the *DELLA* genes were vertically inherited from *I. aquatica*. The inheritance of genes resulted in the formation of three groups of syntenic gene pairs, which maintained a constant number of *DELLA* genes without undergoing segmental duplication across various *Ipomoea* species. The phylogenetic tree topology reveals that *InilDELLA3*, *InilDELLA5*, and *InilDELLA2* in *I. nil* are clustered within the same group. Nevertheless, *InilDELLA3* and *InilDELLA5* lack syntenic relationships with *DELLA* genes with other species, indicating that *InilDELLA3* and *InilDELLA5* may have originated from *InilDELLA2* via alternative replication mechanisms. Despite gene rearrangements occurring during *DELLA* gene evolution, these positional alterations have not resulted in the loss of syntenic relationships, thereby underscoring the evolutionary stability of the *DELLA* gene within the *Ipomoea* genus. An examination of the physicochemical properties, gene structure, and protein structure of *DELLA* genes within the genus *Ipomoea* reveals a high degree of diversity both among and within species. In addition, the varying expression profiles observed in sweet potato suggest that *DELLA* genes may have a role in regulating gene expression in either the above-ground or below-ground parts of the plant and in responding to abiotic stresses in sweet potato. Analysis of *cis*-acting elements provides further evidence of species-specific differences in the regulation of *DELLA* genes. A comprehensive understanding of the broader implications of these genes in growth, development, and stress responses facilitates further exploration of the *DELLA* gene family, particularly in terms of its evolutionary history and biological functions. These findings are of significant value to sweet potato breeders seeking to develop transgenic crops that are resilient to a range of conditions and environmental stresses.

## Data Availability

The original contributions presented in the study are included in the article/**Supplementary Material**. Further inquiries can be directed to the corresponding author.

## Supplementary material

Supplementary Figure 1 3-D structure of DELLA proteins predicted by AlphaFold3 in six *Ipomoea* species. **(A-F)** 3-D structure of DELLA proteins of *I. aquatica, I. batatas, I. cairica, I. trifida, I. triloba*, and *I. nil.* The DELLA domain is highlighted in green.

Supplementary Figure 2 Hierarchical clustering tree of genome-wide proteins in sweet potato. The hierarchical clustering tree of whole-genome proteins in sweet potato, clustered by the STRING database based on functional associations between proteins. The clusters are divided into parent clusters (in green) and subclusters (in blue). By analyzing the hierarchical structure, broader functional modules and their submodules can be identified, such as recognizing modules with broad functions at higher levels and specific functional submodules at lower levels. Each dot represents a cluster.
