# Supplementary figures and images for: Evolutionary analysis of DELLA proteins in sweet potato and related species reveals their roles in development and stress responses
Source: Front Plant Sci. 2025 Jan 23;16:1494621. doi: 10.3389/fpls.2025.1494621 (PMC11798988; doi:10.3389/fpls.2025.1494621)

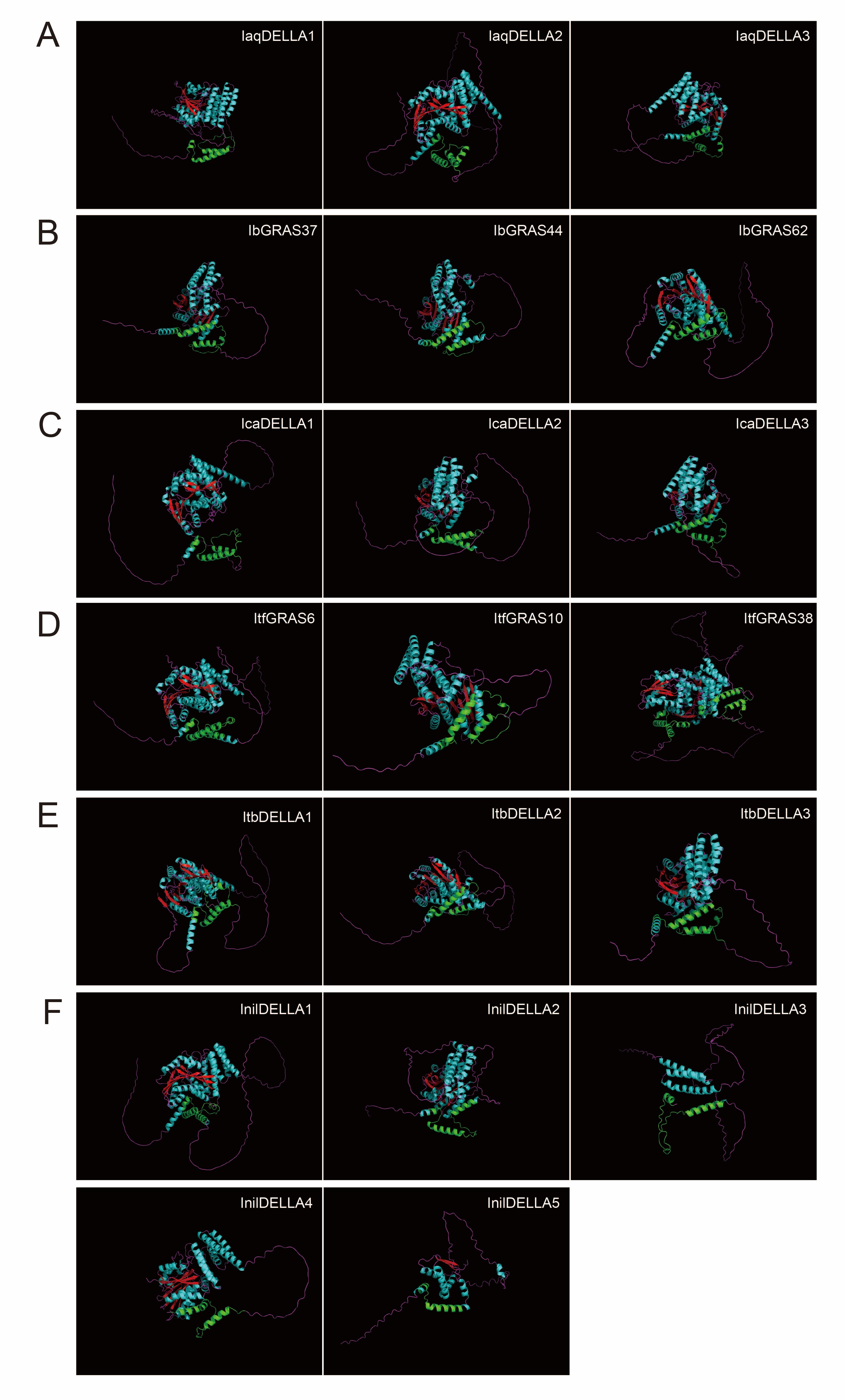

Supplement: Supplementary Figure 1 — 3-D structure of DELLA proteins predicted by AlphaFold3 in six Ipomoea species. (A-F) 3-D structure of DELLA proteins of I. aquatica, I. batatas, I. cairica, I. trifida, I. triloba, and I. nil. The DELLA domain is highlighted in green. [file Image1.jpeg]

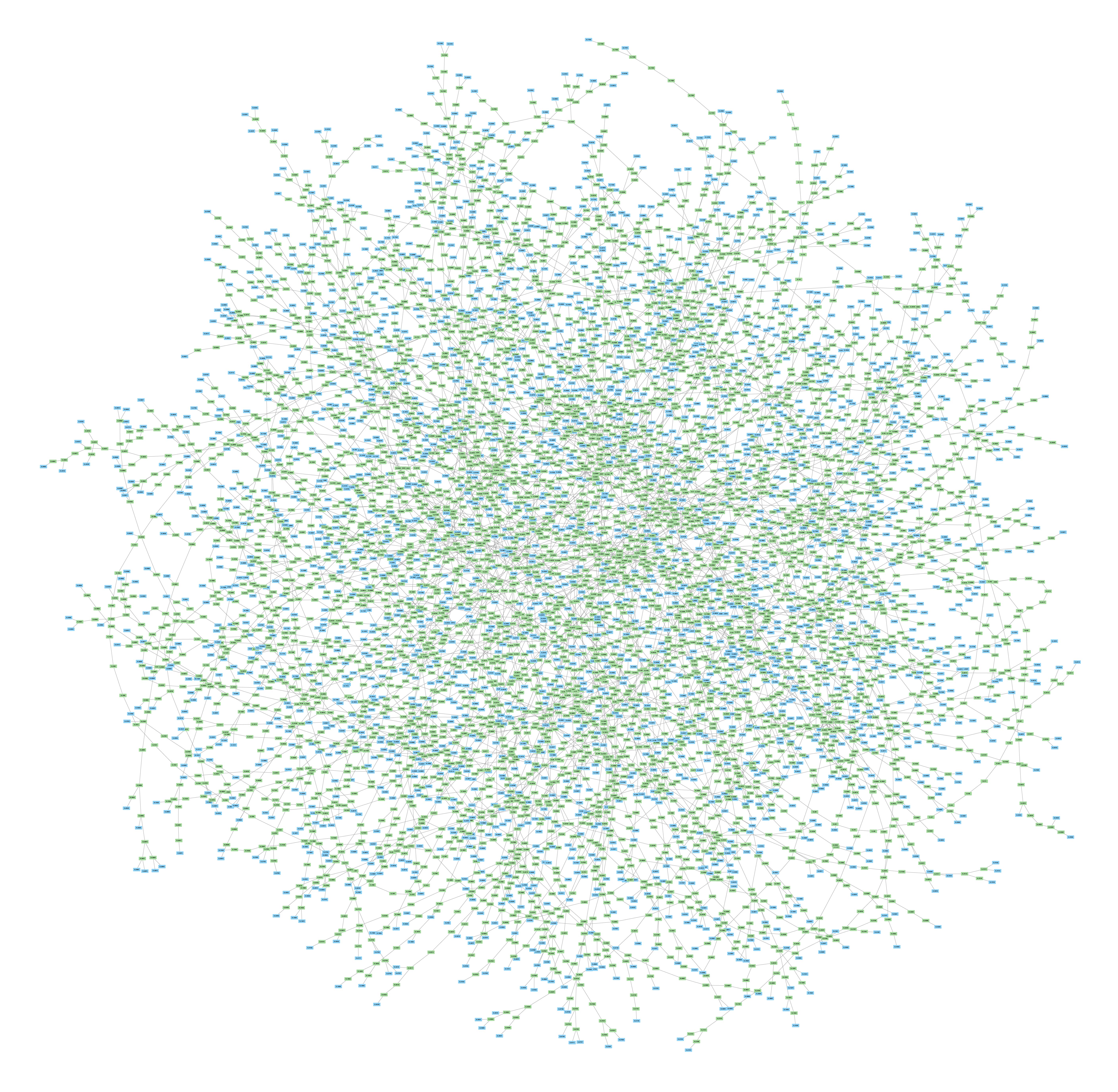

Supplement: Supplementary Figure 2 — Hierarchical clustering tree of genome-wide proteins in sweet potato. The hierarchical clustering tree of whole-genome proteins in sweet potato, clustered by the STRING database based on functional associations between proteins. The clusters are divided into parent clusters (in green) and subclusters (in blue). By analyzing the hierarchical structure, broader functional modules and their submodules can be identified, such as recognizing modules with broad functions at higher levels and specific functional submodules at lower levels. Each dot represents a cluster. [file Image2.jpeg]
